# Are menstrual periods an environmental liability? Period poverty and eco‐feminist bioethics

**DOI:** 10.1111/bioe.13417

**Published:** 2025-04-22

**Authors:** Cristina Richie

**Affiliations:** ^1^ University of Edinburgh Edinburgh UK

**Keywords:** eco‐feminism, eco‐feminist bioethics, feminist bioethics, menstrual hygiene management, period poverty, sustainability

## Abstract

Period poverty has led to many initiatives across the world. In some places, period (or menstrual hygiene management [MHM]) products are free and readily found in restaurants, universities and pubs. However, conversations on mensuration management have also led to discussions on sustainability. One ad in a bathroom states, ‘why not try swapping (from tampons or pads) to a…carbon lowering menstrual cup?’ This begs the question not only how can menstruation management be more sustainable, but also, since it (ostensibly) is not, do females have an ethical obligation to limit, or eliminate, periods for the sake of environmental conservation? This question speaks to deeper themes whereby females are vilified for their bodies (think: blaming the victim in sexual assault; blaming females in the developing world for having ‘too many children’; and blaming females for miscarriages related to high‐risk behaviours). This paper will first offer the background of the period poverty movement. It will then explore salient themes related to gender, ecology, ethics and health through the lenses of eco‐feminism and feminist bioethics. The third task of the paper will be to analyse the implications of environmental ethics on female physical functioning, with specific attention to menstruation: this may be thought of as a case study in the underdeveloped area of eco‐feminist bioethics. After describing the ethical and social issues surrounding sustainability and menstrual hygiene management, the paper, fourth, will return to the question initially raised: the ethical obligation to eliminate menstrual periods for the sake of the environment. The paper will claim that (1) females do not have a special obligation for menstrual elimination based on an environmental rationale, but, like all people, have a general ethical obligation to sustainability in all areas of life, including health care choices. (2) Females retain the right for period elimination for any reason, including environmental reasons.

Period awareness has advanced practices that equalize some physical and economic differences between the sexes by offering free menstrual hygiene products in some places. However, the broader discussion requires serious bioethical evaluation, particularly if there are normative implications for placing additional burdens of sustainability on females, which this paper rejects.^1^

Period poverty^2^ has led to many initiatives across the world. In some places, period (or menstrual hygiene management [MHM]) products are free and readily found in restaurants, universities and pubs. However, conversations on menstruation management have also led to discussions on sustainability. One ad in a bathroom states, ‘why not try swapping (from tampons or pads) to a…carbon lowering menstrual cup?’^3^ This begs the question not only how can menstruation management be more sustainable, but also, since it (ostensibly) is not, do females^4^ have an ethical obligation to limit, or eliminate, periods^5^ for the sake of environmental conservation? This question speaks to deeper themes whereby females are vilified for their bodies (think: blaming the victim in sexual assault; blaming females in the developing world for having ‘too many children’; and blaming females for miscarriages related to high‐risk behaviours).

This paper will first offer the background of the period poverty movement. It will then explore salient aspects related to gender, ecology, ethics and health through the lenses of eco‐feminism^6^ and feminist bioethics.^7^ The third task of the paper will be to analyse the implications of environmental ethics on female physical functioning, with specific attention to menstruation: this may be thought of as a case study in the underdeveloped area of eco‐feminist bioethics.

After describing the ethical and social issues surrounding sustainability and period awareness, the paper, fourth, will return to the question initially raised: the ethical obligation to eliminate periods for the sake of the environment.

The paper will claim that

1. Females do not have a special obligation for menstrual elimination based on an environmental rationale, but, like all people, have a general ethical obligation to sustainability in all areas of life, including health care choices.
2. Females retain the right for period elimination for any reason, including environmental reasons.

Period awareness has advanced practices that equalize some physical and economic differences between the sexes by offering free menstrual products in some places. However, the broader discussion requires nuance and serious bioethical evaluation,^8^ particularly if there are normative implications for placing additional burdens of sustainability on females, which this paper rejects.

## 1 THE PERIOD POVERTY MOVEMENT

One of the earliest uses of the term ‘period poverty’ in English‐speaking literature was 2017.^9^ The term refers to the gap between the cost of menstrual hygiene products and the ability of people who need these products to afford them; period poverty is eliminated through access to affordable (or free) menstrual hygiene management (MHM). The period poverty movement was initially started not from bioethics or public health but (apparently) as a grassroots campaign from students who wanted schools to provide free menstrual products to schoolgirls.^10^ Educators supported the call for free menstrual products in schools and in May 2017, the United Kingdom's National Union of Students (NUS) ‘passed an unprecedented motion committing [Scottish parliament] to providing sanitary products to all female undergraduates in the UK’.^11^

It is interesting to note that before period poverty awareness campaigns ran in the United Kingdom, numerous organizations brought menstrual hygiene products to the developing world as part of humanitarian relief and charitable services. For instance, Amika George notes that the U.K. charity Freedom4Girls ‘which provides menstrual products to women and girls in Kenya, was approached for donations by a school in Leeds, which had become increasingly concerned about the number of girls recurrently absent from school’.^12^

By 2019, the term ‘period poverty’ expanded in scope to include ‘WASH: Water, Sanitation and Hygiene’ facilities, which are also required for menstrual management.^13^ Data from the World Health Bank in 2022 state that, ‘on any given day, more than 300 million women worldwide are menstruating. In total, an estimated 500 million lack access to menstrual products and adequate facilities for menstrual hygiene management (MHM)’.^14^ In the Western world, MHM includes pads, reusable cups, reusable underwear, tampons and menstrual suppression or elimination through menstrual delay tablets or continual use oral contraception (CUOC), inter‐uterine device (IUD) or coil, contraceptive implants or injections or hysterectomy. In the global South, MHM includes use of rags, mud, sticks and other natural, non‐economized resources. Of course, females anywhere in the world might use, or have access to, all or none of these.

By linking period poverty with menstrual hygiene management, economics and public health merged, which gave it a public platform. To be sure, MHM and menstruation is a topic as old as time and has been discussed in academia—in feminist studies^15^—in humanitarian and global health efforts,^16^ and in art.^17^ But, it seems that the catalyst for action over period poverty came from the West and is exported not only internationally^18^ but also domestically.

As of 2024, period poverty refers to a number of interlocking social, biological, economic and ethical aspects of menstruation. For instance, Laura Rossouw and Hana Ross note impacts on health, stating ‘the existing research links unhygienic conditions for using, cleaning and drying MH products to reproductive tract infections’.^19^ They point to social impacts, like sexual exploitation, observing ‘cases of the economically vulnerable [females] having risky transactional sex for sanitary pads’. They continue their assessment by recording ‘how fear and shame around menstrual hygiene as a result of stigmatization inhibit mobility and participation in society, which results in social isolation’ and the impact on ‘the labour market and educational outcomes’ due to absences from being unable to manage the pain of menstruation or the physical aspect of it.^20^ Two other aspects of period poverty that have profound ethical implications are the concomitant sexism and stigma surrounding periods and exclusion ‘from religious and other social activities, any interaction with males, or travelling outside the home’.^21^

Period poverty has a certain a certain cultural cache, with the 2022 WHO statement on menstrual health and rights lauding ‘activists—including young people—and nongovernmental organizations [that] have done much to place menstrual health on the agenda’.^22^ Indeed, menstrual studies include a *Palgrave Handbook of Critical Menstruation Studies*,^23^ a Menstruation Research Network^24^ and non‐university courses.^25^ As noted, MH products are available in some places for free.

Yet, the period poverty movement is not without criticism. These places of ‘free’ access to menstrual products are not really free; they assume a certain level of privilege and physical mobility. Concomitantly, MHM has also adopted a note of elitism through virtue‐signalling. The subtext of the MHM movement indicates that there are environmental contours to MHM, which may be ethically prescriptive as well as descriptive. Period poverty awareness and mitigation have entered another phase in some parts of the Western world.

## 2 ECO‐FEMINISM, FEMINIST BIOETHICS AND PERIOD POVERTY

To be sure, period poverty awareness has done much to highlight, contain and hopefully reverse the stigmas of menstruation and the barriers to social and economic participation that many females face because of menstruation. This paper is not denying the benefits that period poverty awareness has conferred on some females and the potential that it has in the future.

However, this movement has also opened the door to a different sort of stigma: the environmental impact of menstruation. While this is, ostensibly, not the intended outcome, the logical extension of the message has troubling implications.

Alongside free tampons and pads, menstruating females are confronted about how they are negatively affecting the environment. In 2019, Zero Waste Scotland ran a campaign called Trial Period, ‘which offered people in Scotland a free trial of reusable period products’, and raised awareness about the general environmental impact of MH products.^26^ In addition to news coverage in the BBC,^27^ the campaign put posters up across the country in places where it would be effective—such as bathrooms. In some places, the posters were up until 2023; other social media assets are still available.^28^ These designs were in the shape of the reusable pad, pants (i.e., underwear) or cups and read, for instance, ‘why not try swapping to a non drying once a day changing no fuss making money saving ten year lasting carbon lowering reusable menstrual cup?’

Thus, females are confronted with two, possibly contradictory messages in the toilet: that tampons and pads are freely available as part of human rights and that the use of such products has a negative environmental impact. While all people should be interested in lower carbon lifestyles, this message is ethically unique in two ways. First, it reinscribes problematic narratives linking females, ecology and physical capabilities, some of which have been challenged in eco‐feminism and feminist bioethics, and second, seems to require a response from females justifying their current (unsustainable) MHM practices, which are traced back to menstruation itself.

### 2.1 Eco‐feminism

According to Margarita Estévez‐Saá and María Jesús Lorenzo‐Modia, ‘Francoise D'Eaubonne first coined the term ‘ecofeminism’ on the occasion of the publication of *Le Feminisme ou la Mort* in 1974’.^29^ Ecological or Eco‐feminism is a broad and evolving concept but, in its simplest form, examines at the connections between women (the gender), females (the sex) and nature.^30^ This bidirectional study may comment on how, in the first place, women and females can advocate for nature and sustainability, and, in the second place, how women and females may be affected by climate and the natural environment.

On the former point, for instance, the construction of the male‐culture/female‐nature dichotomy has been critically examined.^31^ On the latter point, the environmental effects of the industrialized world on women's bodies—whether largely imposed by environmental practices, as highlighted by Rachel Carson, who exposed the link between DDT in water streams and birth defects of foetuses that females were carrying,^32^—or chosen by women, as in the case of modern medicine, with scholars such as Janice Raymond calling artificial contraception a ‘glaring pollution of women’^33^—are assessed.

This paper does not have the space or scope to detail other connections in eco‐feminism,^34^ but will simply note that period poverty may fit within ecofeminism on these same two points. (This is not to say that literature has discussed these issues, but that it could; they might be areas for future research.)

Thus, first, eco‐feminism might observe that nature and females are connected, in that many or most females have a natural function that includes menstruation and must manage this as part of ‘natural’ biological life; this management has historically been through natural means (cotton; cloths); and menstruation connects to other aspects of biological life and lifecycles. Moreover, environmental factors affect menstruation either by hastening it through hormones in food and water^35^ or by altering it through endocrine disruption.^36^

Second, eco‐feminism might reiterate that the modern means of MHM affect the environment. Everything has a carbon footprint that contributes to climate change and climate change health hazards, loss of biodiversity and other ecological problems. One study from 2022 found that in the United Kingdom, there are 15 million people who menstruate and the ‘estimated menstrual waste accounts for around 28,114 tonnes per year’, with a carbon footprint of 82,602,523 kg per year.^37^ These MHM products range in environmental impact, with the same study calculating that annually, single‐use tampons have a carbon footprint of 0.018 kg; single‐use pads have a carbon footprint of 0.029 kg; reusable underwear has a carbon footprint of 0.11 kg; and reusable cups have a carbon footprint of 0.42 kg.^38^ In addition, a 2023 article from *Environment, Development and Sustainability* conducted a ‘life cycle assessment and environmental cost indicator (ECI) for disposable and reusable pads (virgin cotton, recycled cotton, banana fibers)’^39^ in order to point towards sustainably reducing period poverty and found that of these, ‘disposable pads incur high costs and environmental challenges due to plastic content…banana fiber pads are the most environmentally, economically, and socially viable, with an ECI 7 times lower than disposable pads and 3 times lower than reusable cotton pads’.^40^ They conclude, ‘bio‐based reusable pads can enable local and women‐led production, empowering and providing women with an income source, while ensuring affordable access to sanitary products to combat period poverty’.^41^ This second aspect—the carbon of environmental impact of periods and therefore MH products—will be picked up later in this paper.

### 2.2 Feminist bioethics

Like other interdisciplinary fields, feminist bioethics connects feminist studies and biomedical ethics. Both are relatively recent, Western phenomena. Debora Diniz and Ana Cristina Gonzalez Velez trace the emergence of feminist bioethics to the 1990s.^42^ Of course, feminism as a movement began much earlier—with some pointing to the suffragist movements in the West as the inception of feminism.

Feminist bioethics grew out of a concern that ‘bioethics has largely ignored gender and feminism, long after the rest of the humanities and law have found such work to be important’.^43^ Thus, feminist bioethics draws upon a feminist methodology and epistemology—highlighting commitments like interconnectedness, care, mutuality and relational autonomy^44^—thus bringing feminism/s across to biomedical ethics. Early on, feminist bioethics also showed a particular interest in addressing biomedical and health issues that primarily, or disproportionally, affect female bodies—such as pregnancy, breast cancer^45^ and HIV/AIDS,^46^ before expanding commentary into virtually all areas of biomedical ethics, including euthanasia,^47^ moral enhancement^48^ and moral distress.^49^

Of course, feminism and feminist bioethics are not just for females and women, and this paper does not have the space to detail other connections between feminism and biomedical ethics,^50^ but will simply note that period poverty may be of academic interest in feminist bioethics. This does not mean that articles have been published on the following topics, but that they could be explored, first, in offering a critical commentary on the medicalization of female reproductive functioning, including menstruation and amenorrhoea.

Medicalization has been a well‐discussed topic in modern biomedical ethics since at least 1960^51^ and refers to classifying non‐medical issue, whether physical like ‘too much sleep’,^52^ or mental‐like ‘madness’,^53^ or personal characteristics, like adolescent ‘oppositional defiant disorder’,^54^ within the medical domain.

To be sure, there are health issues associated with unhygienic MH management^55^; menstruation can cause severe pain for some people^56^; and lack of proper MH products can cause mental distress.^57^ Nonetheless, medicalization of the average, normal period is demonstrated by menstrual tracking apps^58^ and creating a diagnostic of ‘menstrual disorders’ that are not associated with pain.^59^ While there are debates about whether medicalization of menstruation helps or harms females, the colonization of the female body by the medical industry bleeds over into period poverty, which itself can be medicalized.^60^ Menstruation is not a pathology, but when it is treated as such by health care, feminist commentary is apt.

A second aspect of interest for feminist bioethics is the management of menstruation as a medical issue. This might take a few different forms. As noted above, there are health issues associated with unhygienic MH management and thus period poverty may require medical attention if there are sequalae from unhygienic MHM practices, in addition to preventive care through proper MH access. Therefore, in addition to feminist bioethics examining the medicalization of menstruation, another form of feminist commentary might assess the medicalization of menstrual management.^61^ As mentioned earlier in the paper, menstruation can be managed through menstrual hygiene or menstrual suppression. The former relies on ‘natural’ means like pads and rags and other ways to absorb or contain menstrual blood and the latter relies on developments of the medical industry.

Primary forms of medical menstrual suppression also double as birth control. For instance, oral contraceptives, when taken continuously, suppress menstruation. Since the advent of the birth control pill, the connection between predicting and regulating menstruation and the ability, therefore, to suppress menstruation have been made. By the 2000s, use of oral contraception was a fairly well‐known and widely used way for physicians to help patients with long, difficult or painful menstrual cycles, with one survey of 900 German gynaecologists stating that 97% ‘had had used [prescribed] a long‐cycle regimen of OCs [oral contraception] for a limited period of time. The reasons were medical indications (menstruation‐related disorders or complaints, endometriosis, polycystic ovary syndrome [PCOS], increased contraceptive efficacy) or request by the woman’.^62^

By 2003, menstrual suppression became commercialized and, with FDA approval, the United States market saw the launch of ‘Seasonale’, the ‘first birth‐control pill specially designed to reduce the frequency of women's periods—from once a month to four times a year’.^63^ In 2015, the drug company Teva Europe launched the ‘extended‐regimen oral contraceptive, Seasonique for the prevention of pregnancy’ for Austria, Italy, Poland and Slovakia with the VP stating ‘this product enables women to change their habits, especially those who do not want to be limited by monthly periods’.^64^ Seasonique had been available in the United States since 2006, and was already on the Brazilian, Chilian and Israeli market. The marketing campaigns above were aimed at females who were already using birth control and wanted the additional choice of limited menstruation. But, of course, once the medical industry has made something available for a clinically indicated reason, like prevention of maternal mortality and morbidity, off‐use and lifestyle use quickly follow.

Currently, ‘period delay tablets’ are sold over the counter in many drugs stores in the United Kingdom after an ‘online consultation’ (i.e., a form that one fills out). Note the branding strategy—this is clearly not, as Seasonique VP stated, ‘a new choice in contraception to achieve greater freedom and confidence in their birth control’,^65^ but, as U.K. drug store Boots advertises, a way to ‘postpone your period, not your plans’.^66^ While I was unable to find data on what percentage of females use which type of contraception to suppress or eliminate menstruation, the discussion in academic journals like *Obstetrics and Gynecology* notes numerous types of birth control that can also be used for menstrual suppression, including ‘combined oral contraceptive pills, combined hormonal patches, vaginal rings, progestin‐only pills, depot medroxyprogesterone acetate, the levonorgestrel‐releasing intrauterine device, and the etonogestrel implant’.^67^ This second aspect—medical menstrual suppression—will be picked up later in this paper.

## 3 ECO‐FEMINIST BIOETHICS AND PERIOD POVERTY

The field of eco‐feminist bioethics is strikingly underdeveloped. Contributions include an article in the Spanish journal *Feminismo/s*, which briefly connects ecofeminist to bioethics^68^; a PhD dissertation on an ‘ecofeminist perspective to empirical (bio)ethics’^69^; and a master's thesis providing ‘An Ecofeminist Analysis of In Vitro Fertilization’.^70^ As with the other, interdisciplinary, feminisms, eco‐feminist bioethics might be envisioned as having an eco‐feminist methodology when approaching biomedical ethics, or may address biomedical issues that more dramatically affect female health from an environmental perspective. The objective of this section is to highlight one—but certainly not the only—eco‐feminist analysis of the period poverty/MHM awareness movement in both methodology and topic.

### 3.1 Eco‐feminist bioethics methodology and period poverty

Menstruation management has an environmental impact. This is a descriptive statement—virtually all human actions impact the natural environment. However, there is often a normative value added to any action that impacts the environment, given the ecological crisis. Yet, the vilification of women's bodies—for any reason, ranging from ‘sexual temptation’ (to heterosexual males), to ecological burden, to inferiority—is a hallmark of sexist society. Females are indeed targeted in menstrual hygiene management in a way males will never be because of biology. Moreover, there is no parallel ecological commentary of a male‐specific body function that has an environmental impact. Thus, females are in a vulnerable position for environmental attack of the impact of menstruation (and, by extension, gestation and breastfeeding; both males and females are environmentally responsible for reproduction).

The ‘othering’ of females reifies rhetoric that places the ‘other’ as inferior, thus creating power imbalances and hierarchies along male/female lines. ‘Other’ need not be indicative of a vertical positioning. However, the message is that there are steps that one group of people can, or should, take to, in this case, minimize environmental impact, without also indicating how the non‐targeted group might support, or hinder, these efforts—for instance, in manufacturing more sustainable period products; in reducing their own impact in other ways; or by facilitating the means to access to sustainable MHM—is prominent. This, in turn, has the troubling ethical implication of unnecessarily creating eco‐shame^71^ and eco‐blame.^72^ The former can be described as the self‐imposed guilty feeling when one participates in an activity or action that is not deemed to be environmentally friendly, whereas the latter is externally imputed and stigmatizing.

Moreover, an eco‐feminist methodology might also point out that, in some cases, research demonstrates that females express concerns over environmental risks more than males,^73^ are more likely to attempt to be ‘be green’^74^ and already more altruistic^75^ and self‐sacrificing^76^ by socialization, not ‘by nature’. This constellation of personality and action might nudge females towards making more dramatic changes in their lives as forms of environmental tradeoffs^77^ if they do not wish to utilize more sustainable forms of MHM, or may nudge females to act more decisively to eliminate the conflict of ‘unsustainable’ MHM directly through menstrual elimination. Neither of the nudges are inherently unethical if done with autonomy and personal choice. However, the socialized nudge to disregard self‐interest has been greatly warned against in feminism and, indeed, bioethics.^78^

### 3.2 Eco‐feminist bioethics and period poverty as a topic

Until the last century, menstruation was something that females had little control over. However, today, females do have control over not only how sustainable their menstruation is but also if they menstruate at all. As noted above, menstrual suppression is available as a lifestyle choice. This poses an interesting consideration in eco‐feminist bioethics, as the carbon impact of a person's biology and medial choices are discussion points in biomedical ethics.

For instance, in 2012, an article *Ethics, Policy and the Environment* explored the idea of genetic engineering to reduce the carbon footprint of future humans by selecting genes that would result in smaller people, who would use fewer resources.^79^ The carbon impact of sexual intercourse, reproductive technologies and reproduction has been discussed in biomedical ethics.^80^ Beyond this, the carbon emissions of numerous medical^81^—and medicalized^82^—procedures have been explored in an effort to decarbonize^83^ the resource‐intensive medical industry.^84^ Since menstruation can be eliminated through medical means, bioethics is thus presented with a standard dilemma: if the medical technology exists, should humans use it^85^ and, in the case of clear environmental (or personal) benefit—the question of *must* it be used.^86^

Doctors, feminists and bioethicists have commented on menstrual suppression. In the medical world, a 2000 editorial in the *Lancet* answered the affirmative to their article's title ‘should monthly menstruation be optional for women?’^87^ In the same year, the journal *Contraception* ran an article stating that ‘women who may derive particular benefit from reduced menstrual frequency include not only those with medical conditions directly caused or aggravated by menses, but also those serving in the military, female athletes, mentally‐retarded women with menstrual hygiene problems, young teens, and perimenopausal women’.^88^

Feminists have examined a variety of perspectives on menstrual suppression. For instance, choice is a theme in feminism, which resonates with the discussion on period elimination.^89^ It has also been noted that a woman who chooses to eliminate her period might feel empowered to discard gender norms around femininity that tie menstruation to ‘being a woman’.^90^ However, in 2006, an article in the journal *Sex Roles* noted that news coverage of menstrual suppression was biased in three significant areas. First, there was a ‘dearth of information about possible long‐term consequences of menstrual suppression through continuous oral contraceptive use’.^91^ The second concern was that alongside the advertised benefit of cost savings from needing fewer menstrual hygiene products, there was no mention of ‘the cost of additional packs of pills, the cost of *Seasonale* or insurance coverage of this and other pills’.^92^ And, finally, the marketing campaigns ‘seemed to capitalize on negative attitudes toward menstruation as a means to promote or market menstrual suppression’.^93^

There has been surprisingly little written on the bioethical aspects of choosing menstrual suppression, but a number of articles on ‘forcing’ menstrual suppression on females with cognitive disabilities have called to mind the atrocious legacy of forced sterilization and the implications of both reversable and irreversible means of menstrual suppression.^94^ Other, much older, ethical commentary has fallen along general lines of the ethics of contraception as being artificial,^95^ or the safety of long‐term contraception,^96^ without reference to menstrual suppression. The answer to the question of whether menstrual suppression is a proper use of medical technology seems to be ‘yes’ by social and medical consensus, with some really significant dissenters raising important ethical issues that lean towards ‘no’.

However, this layer of environmental impact places the standard bioethical dilemma into a new frame. The ethical analysis of menstrual suppression for any reason of the woman's choosing points to the possibility of elective menstrual elimination for ecology as well. Like other medical procedures, the discussion of carbon impact and carbon savings comes to the fore in decision‐making.^97^ Currently, there is debate as to whether environmental sustainability is a valid reason to choose for, or against, medical treatments.^98^ Perhaps doctors would accept this rationale from females as a reason for seeing menstrual suppression, perhaps not. Paternalism in health care, particularly around women's health care^99^ and pro‐environmental medical choices,^100^ is still a barrier to respecting patient autonomy.

Assuming that the environment is a valid reason to seek menstrual suppression, or that no reason is needed to obtain particular methods of menstrual suppression—in keeping with the current understanding of respect for patient autonomy as a positive right^101^—an eco‐feminist bioethics might laud this as a way for females to reduce the carbon impact of MHM (although it would have to be balanced with the carbon impact of the method of menstrual elimination^102^). Or, alternatively, eco‐feminist bioethics might heed this as another way health care has medicalized female bodies, offering a toxic solution (e.g., hormonal contraception) to what was never a problem in the first place (i.e., menstruation.)

Given the trends to endorse pro‐environmental medical behaviours, even to the extent that some forms of previously, widely used, health care are now banned, like the gas desflurane,^103^ the door is open to ask if menstrual suppression might be morally obligatory. For the sake of argument, the next section will explore this idea on the grounds of individual choice and will frame the question as a voluntary, ethical obligation on the part of the individual, which parallels other discussions in ecology, feminism and bioethics about the morality of individual actions.

## 4 IS THERE AN ETHICAL OBLIGATION TO ELIMINATE PERIODS FOR THE SAKE OF THE ENVIRONMENT?

Within the environmental movement, there are certain trends that place behaviours on a spectrum. For this article, I will categorize these as obligatory, desirable or discretionary. For instance, in many people's opinion, divestment from fossil fuels is ethically obligatory, particularly as there are sustainable energy alternatives. Bringing reusable bags to the grocery store is generally regarded as ethically desirable, since it is an easy behaviour that is carbon reducing. Particularly as there are few competing values preventing the action of reusable bags (one value would be the inconvenience of remembering bags) and sometimes an incentive for doing so (e.g., not having to pay for a disposable bag), there is more latitude for choice than divestment, so it is a weaker moral obligation. Discretionary ethical choices often have the most competing values. For instance, reproduction is the most significant way to be sustainable^104^—more so than being vegetarian, eliminating private transportation and eliminating travel *combined*—but because it is so emotionally laden that it is viewed as environmentally discretionary. Discretionary choices also often come with a qualification that others ought not ‘judge’ these choices because they are so personal.

Note that the categorization that I have presented is a spectrum: behaviours can be debated: eliminating red meat from one's diet might be regarded as obligatory by some and discretionary by others. Because of the carbon impact and ethical implications for animal welfare, it is probably more than ethically desirable and might be placed between desirable and obligatory. Also, the view of the category might not be related to the actual ethical assessment. Reproduction is totally bad for the environment, but when placed in another ethical category—it is good for the national defence^105^—the ethical category changes.

In the case of menstruation and ethical choice, there is a two‐fold question. First, where on the ethical spectrum choosing sustainable MHM *products* lies—obligatory, desirable or discretionary. Here, the answer depends on the situation. Choosing, as the campaigns above have suggested, more sustainable MHM might be ethically obligatory if doing so is relatively simple: if, for instance, more sustainable products are widely available, similar in cost and meet the same preferences for convenience, sanitation, etc. as other products. In this way, little extra effort is required for the female. Sustainable MHM might be ethically desirable where unsustainable options remain and there is limited availability in sustainable options. Like reusable grocery bags, this might be a behaviour where sometimes, the more sustainable option is taken and other times, the less sustainable option is taken. More sustainable MHM might be ethically discretionary if the above criteria are not met, that is, if the more sustainable products are difficult to find, more costly or not as convenient/hygienic, etc. and, moreover, they are at odds with some significant value of the female. Because extra efforts would be required, the ethical evaluation moves along the spectrum.

The second question is where on the ethical spectrum choosing sustainable *menstruation*—menstrual suppression—lies. I would argue that this should be viewed as ethically discretionary, based on the above criteria of burden (i.e., medical side effects of menstrual suppression); values of the female; and area of private concern. However, there are three significant ethical points that are tied to menstrual suppression as a discretionary ethical choice for environmental reasons.

1. *Females do not have a special obligation* for menstrual suppression based on environmental rationale, but, like all people, have a general ethical obligation to be sustainable in all areas of life.
  Although biomedicine has created the possibility of menstrual suppression, this need not demand that females make use of it because of the, arguably, extraordinary burdens that it would incur, namely, the consumption of potentially carcinogenic hormonal contraception, or a painful to place IUD; because some females value menstruation; and because it requires extra effort, that is, finding a health care provider to obtain these services, time spent in medical appointments and side effects of medication. In contrast, simply purchasing MHM products has no physical side effects and preserves normal functioning.
  Another reason why menstrual suppression for environmental reasons could not be ethically obligatory is because a normative obligation entails a duty to act on this obligation. Put another way, an obligation towards menstrual suppression might lead to coercive policies. There needs to be a firm reinforcement in biomedical ethics of the right to autonomy, understood as the negative right to non‐interference and a positive right to choose menstrual suppression or not. Ethically obligatory menstrual suppression obviously could not be maintained without gross violation of female and human rights.
2. *Females retain the right for menstrual suppression* for any reason, including environmental reasons.

Whether or not a female views menstruation as a positive, negative or neutral even is highly variable. Factors include social discourse, cultural factors and individual perspective. Social discourse can be influenced by economic factors, such as the marketing of oral pills for the ‘inconvenience’ of menstruation.^106^ Cultural factors may be tied to notions of naturalness, femininity and education of menstrual suppression.^107^ Individual perspective can be based on activity level, duration of menstruation, cycle intervals, intensity of premenstrual syndrome and feminist commitments.^108^ Social, cultural and individual factors are intertwined and the perception of a particular menses during a particular cycle by an individual is dynamic and highly contextual.

Even so, studies show that continuous use contraception is beneficial to females for both pregnancy prevention and menstrual suppression.^109^ A female may choose this for a variety of reasons, from challenging social situations, demanding vocations, environmental commitments or medical problems associated with menstruation.

First, females with challenging social situations may wish to eliminate menstruation. These situations include females who have gender dysphoria and may benefit from not having a monthly confrontation with their physical menstruation.^110^ By eliminating menstruation, gender harmony is supported. Because of hygiene concerns related to unstable living accommodations—like homelessness^111^—menstrual suppression might be desirable as well. However, addressing the basic need for stable housing is the first concern. Females in long‐term situations of duress, such as female hajis,^112^ are another group that might find menstrual suppression desirable for social reasons.

Second, females in demanding vocations might opt for menstrual suppression. Studies have shown that in the United States, military ‘deployed women report that menses were difficult to manage because military gear affected self‐care and environmental factors including heat, sand, limited restroom facilities, and long work hours without a break [which] prevented changing sanitary products’.^113^ Clearly, menstruation hampers the ability to serve effectively and efficiently. Similarly, female astronauts^114^ and female aviators^115^ might make use of menstrual suppression during missions.

Third, females who feel that disposable or reusable MHM are unsustainable might opt for menstrual suppression. If they do so, they might consider the method used. Pharmaceuticals are incredibly carbon‐intensive^116^ and while data do not exist on the carbon footprint of the various forms of birth‐control pharmaceuticals,^117^ some commonsense assessments can be made. While IUDs/coils do have hormones in them, because of frequency of use (i.e., once every 5+ years) and because of function (i.e., to prevent pregnancy both through suppressing ovulation and interfering with implantation), they are, likely, less resources than contraceptive pills. However, even pharmacological management of menstruation is better for the environment than (accidental or intended) procreation; closing off menstruation also means refraining from pregnancy, so the overall environmental balance should be evaluated.

Fourth, as noted, there are a variety of medical problems that can be associated with menstruation. Menstrual suppression in these cases requires discernment if it is the female's request or the family's. IUDs have historically been used to suppress menstruation in cases of menorrhagia^118^ and hysterectomies are also offered as a long‐term solution to burdensome and incapacitating menstrual cycles rather than merely suppressing menstruation until menopause.^119^

However, both IUDs and hysterectomies have been used in adult females with cognitive disabilities who may not be able to consent.^120^ In one of the most controversial cases, a ‘severely disabled young girl…parents opted for oestrogen therapy, a hysterectomy and breast removal—the so‐called ‘Ashley treatment’.^121^ Thus, choice for females remains central, as do standards of informed consent for both minors^122^ and those with limited capacity to consent.^123^

3. *Both menstrual products and menstrual suppression methods have an ethical obligation‐* based on the discussion above‐ to be more sustainable. This obligation comes from the criteria of burden. To be sure, all of areas of life must simultaneously become sustainable because of the environmental impact of anthropogenic activity. Therefore, it would be misleading to say that manufactures, producers and suppliers of MHM products and techniques have an obligation first to be more sustainable.

Individual responsibility and institutional/social responsibility are not mutually exclusive and should not be consecutive, but concurrent, responsibilities. The focus here has been on individual action, as it is often quicker in effect, since it does not rely on systemic change (think also of other pro‐environmental, individual‐level actions like opting out of procreation; avoiding red meat; and not flying). Individuals might choose sustainability even if the stronger obligation for sustainability falls on business, corporations and governments ‘first’. This should not foster resentment as individuals are moral agents who make their own decisions; they are not forced to make these choices, even if they have very compelling reasons from outside (not within) to make these choices.

Moreover, moral actions by individuals—in alignment with Kohlberg's Stages of Moral Development^124^—are formed and executed independently of others. It might be nice if ethical action happened in a logical sequence that made ethical choices easier for everyone (i.e., reducing food packaging from companies and taxing highly packaged foods), which leads to consumer choices being simple and ethical) but because of the environmental crisis and the task of individual responsibility, the order of moral obligation for sustainability is less important than the imperative for moral responsibility.

## 5 CONCLUSION

Period awareness has advanced practices that equalize some physical and economic differences between the sexes by offering free menstrual products in some places. However, it also raises interesting questions for eco‐feminist bioethics. Likely, the goal of sustainable MHM and menstruation is not to neuter women, or place extra environmental burdens on them, but rather to eradicate the socially imposed boundaries and restriction that have been placed on females from without. This may include more sustainable MHM but it also may include menstrual suppression. The choice, as always, ought to be a free choice, not only free from rhetoric and coercion but also free from environmentally hazardous products and practices both inside and outside of our bodies.

## Footnotes

1 This paper will reference those with a biological sex as ‘female’ and those with a biological sex as ‘male’ for accuracy. The terms ‘woman’ and ‘man’ refer to gender, which is socially constructed and not chromosomal. Although both academic literature and society are suffering from a misunderstanding of the difference between sex and gender—often conflating the two or using them interchangeably—linguistic accuracy, particularly in discussing biology, must be the standard, even if in common parlance there is rhetorical sloppiness. Direct quotes and paraphrases will be stated as written, even if inaccurate. Richie, C. (2019). Sex, not gender. A plea for accuracy. *Experimental & Molecular Medicine*, *51*(11), 1.

2 Boyers, M., Garikipati, S., Biggane, A., Douglas, E., Hawkes, N., Kiely, C., Giddings, C., Kelly, J., Exley, D., Phillips‐Howard, P. A., & Mason, L. (2022). Period poverty: The perceptions and experiences of impoverished women living in an inner‐city area of Northwest England. *PLoS One*, *17*(7), e0269341.

3 Zero Waste Scotland. (2023, April 14). *Trial period*. https://www.zerowastescotland.org.uk/resources/trial-period

4 This paper will reference those with a biological sex as ‘female’ and those with a biological sex as ‘male’ for accuracy. The terms ‘woman’ and ‘man’ refer to gender, which is socially constructed and not chromosomal. Although both academic literature and society are suffering from a misunderstanding of the difference between sex and gender—often conflating the two or using them interchangeably—linguistic accuracy, particularly in medicine, which affects the sexes differently, must be the standard, even if in common parlance there is rhetorical sloppiness. Author (2019).

5 Zero Waste Scotland, op. cit. note 3; See also Zero Waste Scotland. (2023, April 14). *Trial period campaign 2019*. https://www.zerowastescotland.org.uk/trial-period/campaign

6 Johnston‐Robledo, I., Jessica, B., & Stephanie, W. (2006). “Kiss your period good‐bye”: Menstrual suppression in the popular press. *Sex Roles*, *54*, 353–360.

7 Kings, A. E. Intersectionality and the changing face of ecofeminism. *Ethics and the Environment*, *22*(1), 63–87; Norgaard, K. M. (1999). Moon phases, menstrual cycles, and Mother Earth: The construction of a special relationship between women and nature. *Ethics & the Environment*, *4*(2), 197–209.

8 Gross, M. S., Amelia, H., & Bethany, C. (2021). Pay no attention to that man behind the curtain: An ethical analysis of the monetization of menstruation app data. *IJFAB: International Journal of Feminist Approaches to Bioethics*, *14*(2), 144–156; Gunson, J. S. (2012). Menstrual suppression: The rhetoric and realities of choice. In *Outskirts: Feminisms along the edge* (Vol. 27). University of Western Australia, Women's Studies. https://www.outskirts.arts.uwa.edu.au/volumes/volume-27/jessica-shipman-gunson

9 Coryton, L. (2017). UK schoolgirls dealing with period poverty. *Education Journal*, *312*, 26–27. https://web.p.ebscohost.com/ehost/pdfviewer/pdfviewer?vid=1&sid=606843c6-6b89-43a0-bf71-9d2ee96ee960%40redis

10 Started by Amika, G. (2017, March 31). Free menstrual products for all children on free school meals #FreePeriods. Petition to Damien Hinds MP (Secretary of State for Education) and Theresa May MP, Prime Minister of the United Kingdom and Leader of the Conservative Party *Change.org*. https://www.change.org/p/theresa-may-mp-free-menstrual-products-for-all-children-on-free-school-meals-freeperiods

11 Coryton, op. cit. note 9.

12 Started by Amika, op. cit. note 10.

13 Tull, K. (2019). *Period poverty impact on the economic empowerment of women* (K4D Helpdesk Report 536). Institute of Development Studies.

14 Ibid.

15 van der Heijden, J. E. (2020). *Period warriors on Instagram Popular Feminism, Digital Activism & Period Poverty* (Master's thesis, Utrecht University, NL). https://studenttheses.uu.nl/handle/20.500.12932/36546

16 Patel, K., Panda, N., Sahoo, K. C., Saxena, S., Chouhan, N. S., Singh, P., Ghosh, U., & Panda, B. (2022). A systematic review of menstrual hygiene management (MHM) during humanitarian crises and/or emergencies in low‐and middle‐income countries. *Frontiers in Public Health*, *10*, 1018092.

17 Julia, M. (2016, December 12). Let it bleed—Art's revival of menstrual blood. *The Guardian*. https://www.theguardian.com/lifeandstyle/2016/dec/12/let-it-bleed-arts-revival-of-menstrual-blood

18 Chris, B. (2019). *The managed body: Developing girls and menstrual health in the Global South*. Springer.

19 Rossouw, L., & Hana, R. (2021). Understanding period poverty: Socio‐economic inequalities in menstrual hygiene management in eight low‐and middle‐income countries. *International Journal of Environmental Research and Public Health*, *18*(5), 2571.

20 Ibid.

21 Tull, op. cit. note 13, p. 26.

22 WHO Statement on Menstrual Health and Rights, 22 June 2022, at https://www.who.int/news/item/22-06-2022-who-statement-on-menstrual-health-and-rights

23 Bobel, C., Inga, T. W., Breanne, F., Katie, A. H., Elizabeth, A. K., & Tomi‐Ann, R. (2020). *The Palgrave handbook of critical menstruation studies*. Springer.

24 Menstruation Research Network. (2022). Welcome. https://menstruationresearchnetwork.org.uk/

25 Lara, O. (2022). *Contemporary menstrual studies*. https://laraowen.com/contemporary-menstrual-studies

26 Zero Waste Scotland. (2023, November 29). *Trial Period Campaign Assets.* https://www.zerowastescotland.org.uk/resources/trial-period-campaign-assets

27 BBC News. (2019, November 28). Menstrual cups: Stemming the flow of single‐use period waste in Scotland. *BBC News*. https://www.bbc.co.uk/news/uk-scotland-edinburgh-east-fife-50572459

28 Zero Waste Scotland, op. cit. note 26.

29 Estévez‐Saá, M., & Lorenzo‐Modia, M. J. (2018). The ethics and aesthetics of eco‐caring: Contemporary debates on ecofeminism(s). *Women's Studies*, *47*(2), 123–146.

30 Warren, K. J. (1994). *Ecological feminism*. Routledge.

31 Papić, Ž. (1995). Problem odnosa priroda‐kultura i mogućnost zasnivanja antropologije (epistemološke) prošlosti. *Sociologija*, *37*(3), 277–300.

32 Rachel, C. (1962). *Silent spring*. Houghton Mifflin.

33 Janice, R. (1993). *Women as wombs* (p. 134). Harper Collins.

34 A fuller definition indicates the range here: ‘Ecofeminism, in broad terms, has been conceived as both a theory and a movement that associates women and the environment; that describes the connections that throughout history have been established between women and nature from cultural, historical, psychological, spiritual, or political perspectives; that denounces the comparable degradation, subjection, and exploitation of women, nature, non‐human animals, and other marginalized social groups; and one that proposes diverse alternative solutions, addressing both gender and ecological vindications and hence trying to put an end to the violence exerted on women and the underprivileged, as well as to the destruction of natural resources and the extinction of non‐human animals and species’. Estévez‐Saá & Lorenzo‐Modia, op, cit. note 29.

35 Vasiliu, O., Muttinemi, J., & Karmaus, W. (2004). In utero exposure to organochlorines and age at menarche. *Human Reproduction, 19*, 1506–1512.

36 Willem, O., Maribel, A., Pauliina, D., Linda, G., Niels, E. S., Miguel, A. C., Rémi, B., Nicolas, G., Karin, S. H., Rune, L.‐J., Olwenn, M., Jeanne, P., Cristina, R., & Nathalie, V. (2024, March). *Factsheet on environmental exposure and female reproductive health*. European Society of Human Reproduction and Embryology Expert Panel for Evironmental Factors and Fertility. pp. 1–4 at 2. https://www.eshre.eu/-/media/sitecore-files/Factsheets/FACT-SHEET-Female-2024_03_04.pdf

37 Blair, L. A. G., Bajón‐Fernández, Y., & Villa, R. (2022). An exploratory study of the impact and potential of menstrual hygiene management waste in the UK. *Cleaner Engineering and Technology*, *7*, 100435.

38 Ibid.

39 Aridi, R., & Yehya, A. (2023). Sustainability assessment of sanitary pad solutions to reduce period poverty. *Environment, Development and Sustainability*. https://doi.org/10.1007/s10668-023-04338-y

40 Ibid.

41 Ibid.

42 Diniz, D., & Velez, A. C. G. (2018). Feminist bioethics: The emergence of the oppressed. In R. P. Tong (Ed.), *Globalizing feminist bioethics: Crosscultural perspectives* (pp. 62–72). Routledge (Original work published 2000).

43 Wolf, S. M. (1996). Introduction: Gender and feminism in bioethics. In S. M. Wolf (ed.), *Feminism & bioethics: beyond reproduction* (pp. 3–43). Oxford University Press.

44 Jennings, B. (2018). Solidarity and care as relational practices. *Bioethics*, *32*(9), 553–561.

45 Nielsen, E. V. L. (2013). *Disruptive breast cancer narratives: Shaping cultural politics, informing feminist bioethics and performing repair* (PhD diss., University of British Columbia).

46 Wolf, S. M. (Ed.). (1996). *Feminism & bioethics: Beyond reproduction*. Oxford University Press.

47 Bender, L., (1992). A feminist analysis of physician‐assisted dying and voluntary active euthanasia. *Tennessee Law Review*, *59*(3), 519–546.

48 Szocik, K. (2023). Why moral bioenhancement in future space missions may not be a good idea: The perspective of feminist bioethics of space exploration. *Technology in Society*, *75*, 102365.

49 Morley, G., Bradbury‐Jones, C., & Ives, J. (2021). Reasons to redefine moral distress: A feminist empirical bioethics analysis. *Bioethics*, *35*(1), 61–71.

50 Rogers, W. A., Scully, J. L., Carter, S. M., Entwistle, V. A., & Mills, C. (Eds.). (2023). *The Routledge handbook of feminist bioethics*. Routledge.

51 Szasz, T. (1960). The myth of mental illness. *American Psychologist*, *15*, 113–118.

52 Keating, A. (2021). I need a nap: Living with idiopathic hypersomnia in sleepless capitalism. *New Sociology: Journal of Critical Praxis*, *2(*1). https://doi.org/10.25071/2563-3694.68

53 Foucault, M. (1983). *Madness and civilization*. Routledge (French original 1961).

54 Barnhart, M. (2018). Oppositional defiant disorder: The psy apparatuses and youth resistance. *Journal of Progressive Human Services*, *29*(1), 6–27.

55 Torondel, B., Sinha, S., Mohanty, J. R., Swain, T., Sahoo, P., Panda, B., & Nayak, A. (2018). Association between unhygienic menstrual management practices and prevalence of lower reproductive tract infections: A hospital‐based cross‐sectional study in Odisha, India. *BMC Infectious Diseases*, *18*, 1–12.

56 Myszko, O., Al‐Husayni, N., & Talib, H. J. (2020). Painful periods in the adolescent girl. *Pediatric Annals*, *49*(4), e176–e182.

57 Tanton, C., Nakuya, K., Kansiime, C., Hytti, L., Torondel, B., Francis, S.C., Namirembe, P., Nakalema, S., Nalugya, R., Musoke, S.N., & Neema, S. (2021). Menstrual characteristics, menstrual anxiety and school attendance among adolescents in Uganda: A longitudinal study. *BMC Women's Health*, *21*(1), 410.

58 Polzer, J., Sui, A., Ge, K., & Cayen, L. (2022). Empowerment through participatory surveillance? Menstrual and fertility self‐tracking apps as postfeminist biopedagogies. In J. Fellows, & L. Smit (Eds.), *Gender, Sex, and Tech* (pp. 163–184).

59 Barnack‐Tavlaris, J. (2015). The medicalization of the menstrual cycle: Menstruation as a disorder. In M. McHugh, & J. Chrisler (Eds.), *The wrong prescription for women: How medicine and media create a “need” for treatments, drugs, and surgery* (pp. 61–75).

60 Lahiri‐Dutt, K. (2015). Medicalising menstruation: A feminist critique of the political economy of menstrual hygiene management in South Asia. *Gender, Place & Culture*, *22*(8), 1158–1176.

61 Gunson, J. S. (2010). “More natural but less normal”: Reconsidering medicalisation and agency through women's accounts of menstrual suppression. *Social Science & Medicine*, *71*(7), 1324–1331.

62 Wiegratz, I., Hommel, H. H., Zimmermann, T., & Kuhl, H. (2004). Attitudes of German women and gynecologists towards long‐cycle treatment with oral contraceptives. *Contraception*, *69*, 37–42.

63 Associated Press. (2003, September 5). FDA approves new Seasonale birth‐control pill. *NBC News*. https://www.nbcnews.com/id/wbna3077096https://www.nbcnews.com/id/wbna3077096

64 Victoria, W. (2015, July 1). Teva launches Seasonique extended‐regimen contraceptive in first European markets. *European Pharmaceutical Review*. https://www.europeanpharmaceuticalreview.com/news/32959/teva-launches-seasonique-extended-regimen-contraceptive-in-first-european-markets/

65 Ibid.

66 Boots. (2024). *Period delay tablets*. https://onlinedoctor.boots.com/period-delay

67 Adeyemi‐Fowode, O., & Stambough, K. C. (2022). General Approaches to Medical Management of Menstrual Suppression. *Obstetrics and Gynecology*, *140*(3), 528–541.

68 Rey Torrijos, E. (2013). Ecofeminist visions: recent developments and their contribution to the future of feminism. *Feminismo/s*, *22*, 17–46.

69 Cook, J. (2021). *At the intersection: bringing an ecofeminist perspective to empirical (bio) ethics and equity in international research* (PhD diss., University of Central Lancashire).

70 Legal, C. (2015). *An ecofeminist analysis of in vitro fertilization* (MA thesis, University of Toronto).

71 Nielsen, R. S., Gamborg, C., & Lund, T. B. (2024). Eco‐guilt and eco‐shame in everyday life: An exploratory study of the experiences, triggers, and reactions. *Frontiers in Sustainability*, *5*, 1357656.

72 Luke, J. S. (1992). Applied behavioral science in the 1990s: Looking outward more, inward less. *Public Administration Quarterly*, *16*, 222–234.

73 Subiza‐Pérez, M., Marina, L. S., Irizar, A., Gallastegi, M., Anabitarte, A., Urbieta, N., Babarro, I., Molinuevo, A., Vozmediano, L., & Ibarluzea, J. (2020). Who feels a greater environmental risk? Women, younger adults and pro‐environmentally friendly people express higher concerns about a set of environmental exposures. *Environmental Research*, *181*, 108918.

74 Trelohan, M. (2022). Do women engage in pro‐environmental behaviours in the public sphere due to social expectations? The effects of social norm‐based persuasive messages. *VOLUNTAS: International Journal of Voluntary and Nonprofit Organizations*, *33*(1), 134–148.

75 Simmons, W. O., & Emanuele, R. (2007). Male‐female giving differentials: are women more altruistic? *Journal of Economic Studies*, *34*(6), 534–550.

76 Marody, M., & Giza‐Poleszczuk, A. (2000). Changing images of identity in Poland: From the self‐sacrificing to the self‐investing woman. In G. Kligman, & G. Susan (Eds.), *Reproducing gender: Politics, publics and everyday life after socialism* (pp. 151–175).

77 Aschemann‐Witzel, J. (2015). Consumer perception and trends about health and sustainability: Trade‐offs and synergies of two pivotal issues. *Current Opinion in Food Science*, *3*, 6–10.

78 Raymond, J. G. (1990). Reproductive gifts and gift giving: The altruistic woman. *Hastings Center Report*, *20*(6), 7–11; George, K., 2007. A woman's choice? The gendered risks of voluntary euthanasia and physician‐assisted suicide. *Medical Law Review*, *15*(1), 1‐33.

79 Matthew, L., Sandberg, A., & Roache, R. (2012). Human engineering and climate change. *Ethics, Policy and the Environment*, *15*(2), 206–221.

80 Richie, C. (2024). *Environmental ethics and medical reproduction*. Oxford University Press.

81 Rizan, C., Steinbach, I., Nicholson, R., Lillywhite, R., Reed, M., & Bhutta, M. F. (2020). The carbon footprint of surgical operations: a systematic review. *Annals of Surgery*, 272(6), 986–995.

82 Spil, N. A., van Nieuwenhuizen, K. E., Rowe, R., Thornton, J. G., Murphy, E., Verheijen, E., Shelton, C. L., Heazell, A. E. P. (2024). The carbon footprint of different modes of birth in the UK and the Netherlands: An exploratory study using life cycle assessment. *BJOG: An International Journal of Obstetrics & Gynaecology*, *131*(5), 568–578.

83 Husain, S. A., & Sidhu, M. (2021). Realist evaluation of the implementation and impact of the NHS carbon reduction strategy in the UK. *BMJ Open*, *11*(9), e044259.

84 Pichler, P.‐P., Jaccard, I. S., Weisz, U., & Weisz, H. (2019). International comparison of health care carbon footprints. *Environmental Research Letters*, *14*(6), 064004.

85 This is a concern in germline editing. For instance Evans, J. H. (2021). Setting ethical limits on human gene editing after the fall of the somatic/germline barrier. *Proceedings of the National Academy of Sciences of the United States of America*, *118*(22), e2004837117.

86 This is a concern in germline editing. Gyngell, C., Douglas, T., & Savulescu, J. (2017). The ethics of germline gene editing. *Journal of Applied Philosophy*, *34*(4), 498–513.

87 Thomas, S. L., & Ellertson, C. (2000). Nuisance or natural and healthy: should monthly menstruation be optional for women? *The Lancet*, *355*(207), 922–924.

88 Kaunitz, A. M. (2000). Menstruation: choosing whether… and when. *Contraception*, *62*(6), 277–284.

89 Gunson, op. cit. note 8. *Gale Academic OneFile*, https://link.gale.com/apps/doc/A314254243/AONE?u=anon~e4e9f785&sid=googleScholar&xid=5bddc766. Accessed 10 May 2024.

90 Jarvis, B. (2013). No to the flow: Rejecting feminine norms and the reproductive imperative through hormonal menstrual suppression.” In B. Fahs, M. L. Dudy, & S. Stage (Eds.), *The moral panics of sexuality* (pp. 205–225). Palgrave Macmillan UK.

91 Johnston‐Robledo, I., et al., op. cit. note 6.

92 Ibid.

93 Ibid.

94 Paransky, O. I., & Zurawin, R. K. (2003). Management of menstrual problems and contraception in adolescents with mental retardation: A medical, legal, and ethical review with new suggested guidelines. *Journal of Pediatric and Adolescent Gynecology*, *16*(4), 223–235.

95 Turner, J. V., & McLindon, L. A. (2018). Bioethical and moral perspectives in human reproductive medicine. *The Linacre Quarterly*, *85*(4), 385–398.

96 Brown, G. F., & Moskowitz, E. H. (2020). Moral and policy issues in long‐acting contraception. In S. Sherwin, & B. Parish (Eds.), *Women, medicine, ethics and the law* (pp. 51–72).

97 Wang, J., Li, S., & He, B. (2020). Chinese physicians’ attitudes toward eco‐directed sustainable prescribing from the perspective of ecopharmacovigilance: A cross‐sectional study. *BMJ Open*, *10*(6), e035502.

98 Richie, C., & Samuel, G. (forthcoming). Who carries the responsibility for health care carbon reduction? An analysis of ethical and logistical challenges to health care carbon reduction at the individual, regional, national, and international level. *Hastings Center Report*, forthcoming.

99 Richie, C. (2013). Voluntary sterilization for childfree women: Understanding patient profiles, evaluating accessibility, examining legislation. *Hastings Center Report*, 43(6), 36–44.

100 Richie, C. (2018). Greening the end of life: Refracting clinical ethics through an ecological prism. In M. Lysaught, & M. McCarthy (Eds.), *Catholic bioethics and social justice: The praxis of US health care in a globalized world* (pp. 129–142). Liturgical Academic Press.

101 Daniel, C. (1992). When self‐determination runs amok. *Hastings Center Report*, *22*(March/April), 52–55.

102 Cohen, E. S., Kringos, D. S., Kouwenberg, L. H., Weiland, N. H. S., Richie, C., Aarts, J. W., & Hehenkamp, W. J. (2025). Patient perspectives on climate friendly healthcare: An exploratory study in obstetrics and gynaecology. Patient Education and Counseling, 130, 108427.

103 Association of Anaesthetists. (2023, January 12). *Joint statement on NHSE's plan to decommission desflurane by early 2024*. https://anaesthetists.org/Home/News-opinion/News/Joint-statement-on-NHSEs-plan-to-decommission-desflurane-by-early-2024.

104 Murtaugh, P. A., & Schlax, M. G. (2009). Reproduction and the carbon legacies of individuals. *Global Environmental Change*, *19*(1), 14–20.

105 Condren, M. (1989). To bear children for the fatherland: Mothers and militarism. In E. Schüssler Fiorenza (Ed.), *The power of naming: A Concilium reader in feminist liberation theology* (pp. 115–123).

106 Erol, M. (2016). Unnecessary bleeding, unnecessary suffering: Normalcy and necessity of menstruation in promoting NovaSure®. *Journal of Gender Studies*, *25*(4), 367–381.

107 Newton, V., & Hoggart, L. (2015). Menstruation and contraception: Social and cultural issues on young women's decision making, *Contraception*, *92*(4), 403.

108 Golub, S. (2017). *Lifting the curse of menstruation: A feminist appraisal of the influence of menstruation on women's lives*. Routledge.

109 US Medicine. (2011). *Menstrual suppression could help deployed women avoid discomfort, inconvenience*. Compendium of Federal Medicine. http://www.usmedicine.com/agencies/department-of-defense-dod/menstrual-suppression-could-help-deployed-women-avoid-discomfort-inconvenience/

110 Kanj, R. V., Conard, L. A. E., & Trotman, G. E. (2016). Menstrual suppression and contraceptive choices in a transgender adolescent and young adult population. *Journal of Pediatric and Adolescent Gynecology*, *29*(2), 201–202.

111 Parrillo, A., & Feller, M. D. E. (2017). Menstrual hygiene plight of homeless women, a public health disgrace, *Rhode Island Medical Journal*, *100*(12), 14–15.

112 Yassaee, F., Shekarriz‐Foumani, R., & Sadeghi, S. (2017). Menstrual adjustment administering hormonal agents: A survey of Iranian pilgrim women during long‐term travel. *Journal of Obstetrics, Gynecology and Cancer Research JOGCR*, *2*(4), 1–5.

113 Goyal, V., Borrero, S., & Schwarz, E. B. (2012). Unintended pregnancy and contraception among active‐duty servicewomen and veterans. *American Journal of Obstetrics and Gynecology*, *206*, 463–469 at 466.

114 Jain, V., & Wotring, V. E. (2016). Medically induced amenorrhea in female astronauts, *NPJ Microgravity*, *2*, 16008.

115 Powell‐Dunford, N., Cuda, A. S., Moore, J. L., Crago, M. S., Deuster, P. A. (2009). Menstrual suppression using oral contraceptives: Survey of deployed female aviation personnel. *Aviation, Space, and Environmental Medicine*, *80*(11), 971–975.

116 Belkhir, L., & Elmeligi, A. (2019). Carbon footprint of the global pharmaceutical industry and relative impact of its major players. *Journal of Cleaner Production*, *214*, 185–194.

117 However, see Birnbach, M., Lehmann, A., Naranjo, E., & Finkbeiner, M. (2020). A condom's footprint‐life cycle assessment of a natural rubber condom. *The International Journal of Life Cycle Assessment*, *25*, 964–979.

118 Andersson, J. K., & Rybo, G. (1990). Levonorgestrel‐releasing intrauterine device in the treatment of menorrhagia. *BJOG: An International Journal of Obstetrics & Gynaecology*, *97*(8), 690–694.

119 Reid, P. C., & Mukri, F. (2005). Trends in number of hysterectomies performed in England for menorrhagia: Examination of health episode statistics, 1989 to 2002‐3. *BMJ*, *330*(7497), 938–939.

120 Hillard, P. J. A. (2012). Menstrual suppression with the levonorgestrel intrauterine system in girls with developmental delay, *Journal of Pediatric and Adolescent Gynecology*, *25*(5), 308–313.

121 Edwards, S. D. (2011). The case of Ashley X. *Clinical Ethics*, *6*(1), 39–44.

122 Boceta, R., Martínez‐Casares, O., & Albert, M. (2021). The informed consent in the mature minor: Understanding and decision‐making capacity. *Anales de Pediatría (English Edition)*, *95*(6), 413–422.

123 Dunn, M., Strnadová, I., Scully, J. L., Hansen, J., Loblinzk, J., Sarfaraz, S., Molnar, C., & Palmer, E. E. (2024). Equitable and accessible informed healthcare consent process for people with intellectual disability: A systematic literature review. *BMJ Quality & Safety*, *33*(5), 328–339.

124 Lawrence, K. (1981). *The philosophy of moral development: Moral stages and the idea of justice*. Harper & Row.

